# Prerequisites for social prescribing in Swedish primary care – stakeholders’ perspectives

**DOI:** 10.1080/02813432.2025.2507272

**Published:** 2025-05-20

**Authors:** Frida Degerstedt, Emil Rapo, Emilia W. E. Viklund, Frida Jonsson, Anna Sofia Lundgren, Ingeborg Nilsson

**Affiliations:** ^a^ Department of Community Medicine and Rehabilitation, Umeå University, Umeå, Sweden; ^b^ Department of Epidemiology and Global Health, Umeå University, Umeå, Sweden; ^c^ Department of Culture and Media Studies, Umeå University, Umeå, Sweden

**Keywords:** Social prescribing, elderly, health promotion, loneliness, primary healthcare, public health

## Abstract

**Background and purpose:**

Loneliness is a complex public health issue that can lead to increased morbidity, with higher prevalence among older adults. Social prescribing may be one way to ease loneliness. This study aims to explore stakeholders’ perceptions of prerequisites for implementing a social prescribing program in a Swedish context.

**Method:**

Reflexive thematic analysis was used to analyse individual semi-structured interviews with eleven stakeholders whose experience were considered relevant for implementing a social prescribing program in the Swedish context. They were selected to provide diverse perspectives related to organisation, position, and geography.

**Results:**

From the analysis three themes were constructed; Where to implement – Necessity to bridge organisational gaps, How to implement – Balancing professional expectations, and For whom to implement – Addressing those with ‘real’ needs. These themes highlight the perceived prerequisites, including barriers and facilitators, for successful implementation of social prescribing.

**Conclusions:**

The participants’ perceptions are suggesting that Sweden has several practical advantages in place for implementing social prescribing, such as robust organisations responsible for citizens’ health and well-being and a range of activities available. If organisations can collaborate by prioritizing patients’ needs and overcoming organisational divisions and responsibilities, there is potential for successfully implementing social prescribing in Sweden in the future. Nevertheless, implementation may be hampered by limited resources within health care, and challenges to evaluate program effects.

## Introduction

Loneliness and its negative health effects, particularly among older adults, has become a growing concern worldwide. Reported prevalence of loneliness among Swedish older adults spans from 17% [1] to as high as 50% [2], with feelings of loneliness varying in intensity over time while typically increasing with age [1]. Loneliness is associated with a range of health problems such as depression, anxiety, eating disorders, Alzheimer’s disease and cognitive impairment [3] as well as with increased mortality [4].

Social relationships are known to be a protective factor for good health [4,5], and efforts are being made to increase social interaction and building new relationships among lonely older adults. Social prescribing has emerged as a strategy to reduce loneliness through connecting patients in primary care with community social activities [6]. Non-controlled evaluations of social prescribing programs have shown short-term benefits, such as reduced loneliness and improved self-rated health and well-being [7–13]. However, controlled studies with longer follow-ups have not showed similar results [14]. Although social prescribing programs have been developed globally, they are most widely implemented and evaluated in the United Kingdom (UK) [15]. While the design and implementation of social prescribing programs varies between contexts, a common feature is a ‘link-worker’ who has an established network of existing social activities within the relevant community and who collaborates with prescribers in primary care [16]. The World Health Organization (WHO) [17] have introduced a toolkit for social prescribing in which adaptations to local contexts and involvement of all relevant stakeholders are advised.

For successful implementation, the target organisation needs to have a readiness for change reflecting available resources but also a motivation and willingness to undertake a novel program [18]. Involving stakeholders that are presumed crucial for implementation, as well as representatives for a community is important in the early stages of an implementation [19,20]. In this study, we focus on stakeholders who have insight into essential prospects in terms of an implementation of a social prescribing program in a Swedish setting.

The aim of this study was to explore stakeholders’ perceptions of prerequisites for implementing a social prescribing program in the Swedish context.

## Materials and methods

The study employed a qualitative design with individual semi-structured interviews analysed using reflexive thematic analysis according to Braun and Clarke [21]. This approach is suitable when the aim of the research is to explore complex views and perceptions.

### Study setting

As part of a research project about social prescribing in a Swedish context [22], this study is set in a tax-financed welfare state with a decentralised health system. The 21 regional councils and 290 municipalities manage their own resources [23]. Primary care is both public and private, where private actors may operate under contract with the National Healthcare Services. Primary care centres, public or private, handle common medical issues and is the first point of contact non-acute health issues. Municipalities have an overall responsibility for the welfare of their citizens, partly on a primary care level, including several aspects of social care and elderly care with support from primary care centres. In the current study, ‘primary care’ refers to these centres.

### Participants and recruitment

Through the research project ‘Social prescribing in Sweden’ [22], relevant stakeholders from different geographic regions in Sweden were identified and recruited to this study through purposive sampling. Inclusion criteria required participants to hold a supervisory or managerial position, involving older adults and loneliness. They were also interested in the topic and could potentially have a role in implementing social prescribing.

In 2020, 18 stakeholders were invited *via* e-mail to participate in individual interviews. The invitations included information about the research project, social prescribing in general, and the interview format and content along with a consent form. One reminder was sent two weeks later.

Eleven stakeholders voluntary agreed to participate, while seven declined due to time constraints or perceived irrelevance. The stakeholders included both men (*n* = 4) and women (*n* = 7), from various professional positions, including management (*n* = 7), co-ordination (*n* = 1), organisational development (*n* = 2), and project management (*n* = 1). They worked in healthcare, municipal services and the voluntary sector, representing both urban and rural areas. The participants did not have prior relations with the interviewer and knew him only by name and occupation.

### Data collection

A semi-structured interview guide (Appendix 1) was constructed and piloted to ensure question clarity. The guide focused on themes such as participant’s views on loneliness, perceptions of social prescribing, experiences with similar programs, and the importance of various support aspects. In line with the qualitative design and reflexive approach, the guide’s questions were alternated with prompts and follow-ups. One on one interviews were conducted by ER, digitally *via* Zoom [24] due to Covid-19 restrictions. Interviews lasted 50 to 60 min. Before starting, the interviewer ensured that the participant had reviewed the study information, addressed any questions, and signed the consent form. The interviews were audio recorded, and transcribed verbatim.

### Data analysis

The data was analysed using reflexive thematic analysis [21] and managed with the MAXQDA software [25]. To ensure that the overall context was not lost, transcripts were read multiple times by the authors. The team, with a background in Ethnology, Public health, social sciences, Developmental psychology, Occupational therapy and Physiotherapy, engaged in frequent discussions to resolve discrepancies. A key principle throughout coding was to keep close to the data and be reflexive of contextual meanings, rather than focusing on the transcripts’ semantics.

Coding was performed by ER and FD and focused on the stakeholders’ perceptions but kept open and continuously discussed among the authors. Codes were grouped by latent meaning to form preliminary themes, constructed with a focus on social prescribing and its potential implementation in Sweden. Through an iterative data analysis with discussions between the authors, the preliminary themes and their names were refined several times to reflect content off the final three themes reported in the results.

### Ethical considerations

The study was approved by the Swedish Ethical Review Authority (2020-00659) and the procedure has followed the Declaration of Helsinki [26]. Although interviews about loneliness and health are sensitive, participants did not discuss their personal health or loneliness.

## Results

Three themes were developed during the analysis, illustrating differing perceptions about the potential implementation of social prescribing in Sweden. The first theme, *Where to implement – Bridging organisational gaps*, includes conflicting perceptions of responsibility allotment and collaboration as well as economic and decision-making requirements. The second theme, *How to implement – Balancing professional expectations,* highlights tensions referring to relief at receiving a potential tool for a significant problem and fear of additional workload and emotional strain. The third theme, *For whom to implement – Addressing those with ‘real’ needs,* emphasizes the importance of centring on the lonely individuals within social prescribing, including challenges in attracting and retaining the most isolated individuals. Through these themes, we abstracted what was perceived as prerequisites for a successful implementation, including barriers and facilitators (Table 1).

**Table 1.** Overview of the three themes that constitute the findings.

Theme 1	Theme 2	Theme 3
Where to implement – Bridging organisational gaps	How to implement – Balancing professional expectations	For whom to implement – Addressing those with ‘real’ needs
This theme includes opinions of allocation of tasks and the need for cooperation between organisations as essential for successfully implementing social prescribing.	This theme includes tensions related to social prescribing implementation, ranging from fears of work overload and reluctance to address loneliness for different reasons, to enthusiasm for gaining a tool to help meet a well-known need.	This theme addresses the challenges of identifying individuals who are isolated, and the importance of support and placing the lonely elderly person at the centre of intervention to ensure a relevant and sustainable process

### Theme 1: Where to implement – Bridging organisational gaps

This first theme illustrates tensions in relation to task allocation and necessary collaboration between involved organisations, if social prescribing were to be implemented in Sweden. Overall, there was an agreement among participants that social prescribing should be integrated into existing organisations, with primary care centres being largely seen as the ‘natural owners’, due to their frequent contact with lonely older adults. Yet, concerns included the ever-expanding role of primary care, insufficiently compensated with required resources, and the low priority of preventative interventions, despite political will as illustrated in the following quote.

…primary care isn’t responsible for everything that happens in its catchment area…/… primary care isn’t a demigod that takes care of everything, it has a limited responsibility for certain types of issues. (Person K)

Most participants agreed that additional resources would be required for implementation. Nevertheless, benefits of doing social prescribing within primary care adjacent to other health care tasks were also mentioned, like during follow-up appointments.

You can often identify people [at the primary care centre] who come in for physical issues. That’s the advantage of primary care, I think. You might go there because you have high blood pressure or something, and then you [health care professional] can take the opportunity. (Person C)

Even though primary care was primarily discussed as potentially responsible for social prescribing, municipalities were also considered, given their overlap in responsibilities. While primary care was seen as improving quality through established routines and evaluations of care, municipalities were valued for their flexibility and existing work with citizens’ well-being.

[The municipality is better] because they have social offices and assistance units, and those who work with older adults, like elderly care, home care, municipal services, housing support, maybe family support, elderly guides, things that are part of the municipal services. (Person C)

The wide range of existing activities within the municipalities, and established cooperation with the voluntary sector was seen as beneficial. The voluntary sector was mentioned as an important ally rather than being the one responsible for social prescribing. As they are not bound by strict rules or administration they could quickly start up or terminate activities depending on the citizens’ needs or interests.

Regardless of the perceived benefits and drawbacks of different responsibility allocations, most agreed that collaboration would be essential.

It’s almost our duty to do that [collaborate] where possible, with taxes as public funds, and I also think their [primary care’s] strength is being able to collaborate. So for me, it was a given, to find ways through collaboration. But it won’t be very easy. (Person A)

Some had positive experiences with primary care and municipal cooperation, while others expressed frustration with strict separations regarding responsibility allocation.

I think it’s the organisational gaps, and I’m almost tired of hearing myself say it. It always comes up somehow that authorities and the public sector try to solve cross-sectoral societal problems on their own. It can’t be solved alone…/… Healthcare needs to get better at collaborating with other organisations in general. (Person J)

Another participant critiqued the common practice of working in ‘organisational silos’ instead of collaborating according to individuals’ needs. Collaboration was perceived as easier in smaller communities due to personal connections but potentially fragile if key individuals left. The importance of collaboration was widely acknowledged, despite debates on its implementation.

### Theme 2: How to implement – Balancing professional expectations

This theme highlights tensions around individual, organisational and societal costs and benefits of implementing social prescribing in a Swedish context. Perceptions and experiences within this theme are mainly centred on implementation within primary care.

Some participants were ambivalent about healthcare’s role in addressing loneliness, noting it is not an illness. Others emphasized shared responsibility for overall health to reduce societal burdens. Addressing loneliness was thought to benefit the healthcare system long-term but would require initial resources, political will, and strategic redirection, all dependent on evidence of effectiveness.

What I think is really important is the effect on the individual. If it’s good and we’re working on the right things…/… then the societal impact will follow naturally. So that’s the most important measure. (Person H)

The social prescribing program was thought to require time to be successful, not only for the lonely individual, but for the management decisions of health care providing resources and assessing evaluations. The need for systematic follow-up and evaluation of social prescribing was emphasized, both during and after implementation. However, evaluating social prescribing programs was seen as challenging as other life events may affect outcomes. There were inconsistencies in the perceptions of potential evaluation methods, and this was mentioned as a source of skepticism.

That’s why I’m a bit skeptical about prescriptions, because how do you follow up on something that’s not so easy to measure? It’s the prescriber who should follow up… Do we have evidence for what we’re doing? How do we follow up on it? When do we follow up on it? (Person K)

Nevertheless, important measures mentioned included individual perceptions of loneliness among patients, prescriber workload, compliance, and statistical and economic evaluations. Opinions varied on the effectiveness of financial management for implementation. Some saw it as beneficial, while others feared it might overshadow other care types. Yet, quality of interventions and internal motivation of providers would remain unclear.

If you’re going to manage from the top, you have to manage with money in [city]… Then you adjust accordingly [as a health care professional]; ‘Yeah, we register this because that’s what they want, and we get paid for it’… But if you’re thinking about working more with quality and improvement, you should start from the bottom. It’s better with an idea that originally comes from the ground level. (Person B)

The suitability of the prescriber was seen as important. Introductory training to enhance and structure the prescription process was emphasized. Legitimized professionals were mentioned as best suited for this role, as their authority could lend credibility to prescriptions and potentially improve compliance. Nevertheless, having a genuine interest in loneliness and actively engaging with lonely individuals was generally viewed as key. Some even suggested that these qualities, along with effective communication and motivational skills for behavioural change, were the only prerequisites needed.

You might need some conversation skills, like a psychiatric nurse or someone who’s worked with addiction or is interested in psychosocial issues. It’s not just about throwing out a question; it’s not that simple when you’re dealing with a lonely person. You need some competence to make it work well. (Person C)

Besides considerations of the actual implementation, positive views on social prescribing were noted, seeing it as a feasible solution to loneliness. However, time constraints and the burden on prescribers were concerns, especially when dealing with sensitive issues.

I think it’s mainly about preparing them [social prescribers] for… that this can be tough. You’ll meet people who don’t have a particularly easy life. You also need to build their motivation to participate because I think compliance is pretty low. (Person I)

Similar concerns were also raised about the burden on the patients when being asked about sensitive matters. Loneliness was also portrayed as difficult for the patient to deal with, or as one of the participants described it: *‘at least as hard as quitting smoking’.* There was agreement that simply matching an activity would not solve the complex problem of loneliness, and providing contact information on a piece of paper would not be sufficient.

They can’t just give you contact info on a piece of paper, in that case you will be alone again, but this time you will be alone with a piece of paper. (Person D)

Overall, while potential long-term public health benefits were noted, there were fears about the additional strain on primary care from implementing social prescribing.

### Theme 3: For whom to implement – Addressing those with ‘real’ needs

This theme encompasses participants’ descriptions of the necessity of putting the lonely older adult in the centre of interventions. Yet, the necessity of addressing challenges in identifying lonely older adults, encouraging participation, and maintaining engagement in social prescribing is also highlighted.

Participants noted that municipalities and civil society organisations offer various social activities but often attract already active older adults rather than those who are lonely. Social prescribing was seen as a promising initiative to reach lonely older adults who are otherwise not engaged. Some participants reflected on stigma as a barrier to admitting loneliness and accepting support.

Loneliness is so stigmatized…/… If you find yourself feeling lonely, it takes a lot of courage to say, ‘I’m lonely, I’ve been left out of the flock, will you let me in?’ (Person A)

The narratives also highlighted concerns that the process of participating in, and maintaining social activities would be a challenging, and potentially frightening labour for individuals who have been withdrawn from social contexts for extended periods.

You also have to be aware that changing behaviour is an effort, and it requires a bit of courage, strength, and energy to dare to do some strange things that I might not have done recently. Basically, I need to do more of what I’ve done too little of to get what I want. (Person D)

Hence, lifestyle changes were seen as hard work, further complicated by psychological conditions like anxiety or depression. There was also an apprehension concerning various obstacles that could occur, in addition to the difficulties of lifestyle change, innate in the concept of loneliness and social disconnectedness.

…you know, there are a thousand obstacles. ‘How do I get there by bus, how do I get down the stairs and do I get up the stairs? Can I hear, can I see, where are the toilets?’ There are so many things like that. (Person D)

Financial constraints for the lonely older adults were among the most frequently raised barriers for social prescribing participation. Other concerns referred to distance and transportation to activities, language barriers, physical accessibility, and fear of falling or of feeling unwelcome. Discrimination and impairment were seen as both causes for, and obstacles of, reducing loneliness, emphasizing the need for support from municipalities, the voluntary sector, and healthcare.

We’re supposed to support, but then the person should be guided out…/… It’s not like they’re going through a treatment like that; it’s more about helping the individual find their own tools to counteract their loneliness issues or find ways to get out of it or prevent it. (Person E)

Participants suggested that social prescribing should include further guidance through the process, with guides or link workers playing a crucial role in picking up the process once the prescriptions were written.

You put together what’s important and relevant, which I don’t exactly know what it is, but to create some kind of guide training… That’s the advantage of community life, we see most things and we’re completely free, like we can do anything. (Person F)

The availability of activities was repeatedly addressed, with a need to find relevant and interesting activities for lonely individuals, activities with a low threshold for inclusion. While rural/urban differences were mentioned, availability of activities was largely seen as unproblematic.

It doesn’t have to be any specific activity; they can join the activities that are available. Maybe someone needs a bit of help to get involved discreetly, or maybe they just need someone to hold their hand the first few times, and that’s what’s needed, but activities are available. (Person A)

Hence, a variety of activities exists and initiating new ones was largely seen as uncomplicated. The complexity and uniqueness of loneliness highlighted the need for social prescribing to accommodate individual needs and wants.

## Discussion

Analysing the interviews revealed stakeholders’ perceptions of prerequisites for implementing a social prescribing program in Sweden. Key themes included organisational collaboration, practical and professional challenges, and the need to centre on patient needs.

Most stakeholders viewed primary care centers as the natural arena for implementing social prescribing but emphasized the importance of collaboration with municipalities and the third sector, aligning with experiences from other programs [27,28].

Among stakeholders, the overall view of social prescribing was positive, with high interest in reducing loneliness. In the UK, readiness for change, or ‘buy-in’, has been key for successful implementation [29]. Organisational readiness for change is an elusive, multi-faceted concept, and may be difficult to predict [30]. While there is a generally positive attitude for lifestyle changing interventions among health professionals in Sweden [18], readiness for change is always specific to the type of change that is in focus [30]. Thus, for a successful implementation in a Swedish context, there must be a readiness specifically for social prescribing. Overall, there was a general perception captured in the results that loneliness among older adults is a complex problem that the public sector should address. This would indicate a readiness as organisations perceived they ‘ought’ to-, or perhaps even ‘want’ to do something about loneliness, which are key motivations for organisational readiness for change according to Weiner [30].

In line with previous findings, the causes of loneliness as well as potential intervention to ameliorate it vary [31,32], indicating there is no one-size-fits-all solution [33]. Solutions should focus on overcoming or eliminating barriers to participation and improving accessibility. Amelioration of accessibility in society overall and increase awareness of its urgency was emphasized, aligning with the goals of the Agenda 2030, of maintaining good health and wellbeing, reducing inequalities, and creating sustainable cities and communities [34].

There was a reasoning in the results suggesting that stakeholders believe that prevention and health promotion should be given a larger focus in Swedish health care. Kardakis [18], describes this in her thesis as a general consensus in Sweden on the national level. Even so, the current state of primary care was seen as requiring healthcare providers to prioritize more urgent health issues, which could challenge implementation of social prescribing. The participants’ criticism of the expanding role of primary care may be a sign of wider tensions between policymakers’ goals and frontline capability. Depending on how the ongoing discourse of the role and responsibility of healthcare develops, initiatives for loneliness such as social prescribing may become de-prioritized within primary care, as has been the case for other healthcare programs in the increasingly fiscally pressured Nordic healthcare systems [35]. Despite these challenges, Sweden’s focus on public health and preventative medicine, along with the Good Quality Local Health Care strategy [36,37], is a trend that has been ongoing within the Nordic healthcare systems for some time [35]. In Sweden, the nationwide undertaking includes making healthcare more person-centred, further promote health and prevent poor health as well as increase cooperation between all relevant actors [36,37]. While some stakeholders perceived ‘organisational silos’ to remain the unsatisfactory praxis, the strategy aims of person-centeredness and integrated primary care, aligns well with the core values of social prescribing.

While loneliness among older adults was seen as important to address, suggesting that there is some readiness for change as described by Weiner [30], this seem to be conditioned on collaboration. Collaboration as a key aspect of successful social prescribing programs, and poor information sharing led to isolation, longer patient waiting times, and disengagement from the program, in the UK context [38]. Another aspect mentioned that may condition change is the view that prescribers need specifically engaged in loneliness and the lonely person. While engagement is likely facilitating, making it a prerequisite may also provide a barrier to implementation if no such person is available.

While mirroring our results with previous literature, similarities are visible as described above, but also differences. For example, the role of family and community were completely absent in the discussions of responsibility in our study, with volunteers only featuring as tertiary. This is a marked difference from studies from the UK, where community and volunteers have a more central role in social prescribing programs [39,40]. Hence, this aspect of social prescribing is yet to be explored in a Swedish context.

Any social prescribing program implemented in Sweden must be structured, yet flexible and person-centred, in accordance with previous research [41,42], although implementing a person-centred program will take time and investment [43]. Considering the participants’ view on loneliness as an important public health problem, and the difficulty of attracting lonely older adults to existing activities, it seems the Swedish context provides good prerequisites for implementation of social prescribing with its’ specific format of linking lonely older adults with social activities in a person-centred manner.

### Strengths and limitations

This study followed the COREQ-checklist where applicable [44]. To facilitate reflexivity to the data, the analysis was discussed with the research team that had a diverse background in different disciplines. The study aimed to achieve a wide variation of perspectives from different contexts in Sweden, with close cooperation and discussion between the authors framing the analysis and findings.

Although participants represented a diverse range of different organisations, in both large urban- and smaller rural communities, they were but a few voices from all the relevant stakeholders in Sweden. The voluntary sector especially represents a wide range of different organisations, who’s representation is insufficient within the current study. While our results constitute a relevant general contribution of perceived facilitators and barriers for implementation of social prescribing in Sweden, it may not be representative to all Swedish contexts. It should be noted that the participants had limited or no information about social prescribing beforehand, thus their views may have been different had they had practical experience working with social prescribing. The written and verbal information provided at the beginning of interviews may have also informed participants’ perspectives.

## Conclusions

According to the perceptions of the participants of the current study, Sweden appears to have the prerequisites, with many practical advantages in place, for implementing social prescribing. There are robust organisations responsible for citizens’ health and well-being, and a range of activities and support available in communities. Provided there is successful collaboration between organisations, with existing patient-centred ways of working prevailing across organisational responsibilities, social prescribing in Sweden seems feasible. There are differing views on the role of primary care in health promotion and disease prevention, despite policies such as Good Quality and Local Healthcare advocating for a more proactive approach. Implementation may be hampered by limited resources within healthcare and challenges in generating statistical evidence on the effectiveness of social prescribing. Finally, loneliness has many causes and no simple solutions. Understanding this while implementing social prescribing in a Swedish context will be important, as successfully managing complex problems requires flexibility, collaboration, and person-centredness.

## Data Availability

The data that support the findings of this study are available from the corresponding author, upon reasonable request.

## Appendix 1: Interview guide

**Table:**

General questions and introduction to the interview	Can you briefly describe your position and organisation? Loneliness has become a topic of discussion in recent public debate, particularly concerning the elderly. What is your experience with this? How do you view loneliness among the elderly? How does it manifest in your daily work? *Key points to consider: Public health perspective, is it a current issue for healthcare? Personal experiences, others’ experiences they’ve heard about, general impressions?* When you read about SAR, tell me what your initial thoughts were. Positive or negative? What problems do you think SAR might solve? (Loneliness, health, costs) *Follow-up questions from relevant areas, possibly move to a different topic if appropriate. REMEMBER TO ASK FOR EXAMPLES OF EVERYTHING!* If you imagine that SAR were to be introduced at primary health centres in Sweden, do you feel there’s anything in particular that should be considered beforehand? How would you know that SAR is working? Can you think of any advantages, off the top of your head, to health centres starting to prescribe social interaction? *Have you noticed a need for something similar in your workplace?* Can you describe any risks associated with introducing SAR? *If the person has experience with similar programmes: Can you describe your experiences working with similar programmes? Positive and negative experiences?*
Questions referring to the potential patient or recipient	Can you describe someone you know or a patient you have been in contact with who might benefit from being referred to SAR? What activities do you think would be suitable for this person/patient to be prescribed within the framework of SAR? *How should the activity be organised? Is staff required?* Which people do you think would not benefit from SAR? *Why not? Do you have experience dealing with this type of person? What was it that didn’t work?* Based on your experience, what do you think people/patients need in terms of support to get started with activities? Based on your experience, what is important to consider when referring people to SAR? *Who should make the referral, in what situation, should anyone be able to make a referral, self-referral? Please elaborate on why.*
Questions referring to different professions and what they need	Patients participating in SAR would encounter staff at several stages of the programme. What do you think are the important skills and qualities for staff working with SAR? *What competencies are needed?* What are your thoughts on some form of training for staff working with SAR? *What kind and how extensive should it be?* Working with a new intervention is, of course, always exciting, but new programmes tend to bring new challenges as well. What do you think will be the biggest challenge for staff when it comes to working with SAR? *Time will likely be a common concern, but make sure to address other potential challenges as well!*
Questions referring to organisation and responsibility	As outlined in the information material, SPiS is a collaboration between primary care, the local council, and voluntary organisations or similar. What are your thoughts on this collaboration? SAR, as a programme, involves several organisations sharing tasks and responsibilities. Can you reflect on how these responsibilities should be divided? *Medical responsibility, responsibility for coordination, responsibility for follow-up, responsibility for ensuring the patient gets to the activity?* Can you think of any potential problems that could arise in the collaboration between organisations? *Distribution of costs? Funding?*
